# GFP-Tagged Protein Detection by Electron Microscopy Using a GBP-APEX Tool in *Drosophila*

**DOI:** 10.3389/fcell.2021.719582

**Published:** 2021-08-12

**Authors:** Fred Bernard, Julie Jouette, Catherine Durieu, Rémi Le Borgne, Antoine Guichet, Sandra Claret

**Affiliations:** ^1^Polarity and Morphogenesis Team, Institut Jacques Monod, CNRS, UMR 7592, University of Paris, Paris, France; ^2^Imagoseine Platform, Institut Jacques Monod, CNRS, UMR 7592, University of Paris, Paris, France

**Keywords:** APEX, nanobody, green fluorescent protein, ovarian follicle, electronic microscopy, GBP, *Drosophila melanogaster*

## Abstract

In cell biology, detection of protein subcellular localizations is often achieved by optical microscopy techniques and more rarely by electron microscopy (EM) despite the greater resolution offered by EM. One of the possible reasons was that protein detection by EM required specific antibodies whereas this need could be circumvented by using fluorescently-tagged proteins in optical microscopy approaches. Recently, the description of a genetically encodable EM tag, the engineered ascorbate peroxidase (APEX), whose activity can be monitored by electron-dense DAB precipitates, has widened the possibilities of specific protein detection in EM. However, this technique still requires the generation of new molecular constructions. Thus, we decided to develop a versatile method that would take advantage of the numerous GFP-tagged proteins already existing and create a tool combining a nanobody anti-GFP (GBP) with APEX. This GBP-APEX tool allows a simple and efficient detection of any GFP fusion proteins without the needs of specific antibodies nor the generation of additional constructions. We have shown the feasibility and efficiency of this method to detect various proteins in *Drosophila* ovarian follicles such as nuclear proteins, proteins associated with endocytic vesicles, plasma membranes or nuclear envelopes. Lastly, we expressed this tool in *Drosophila* with the UAS/GAL4 system that enables spatiotemporal control of the protein detection.

## Introduction

In cell biology studies, protein localization is crucial to understand the cellular functions of proteins and for understanding the dysfunction of proteins in diseases. For years, the technique used for this purpose was immunohistochemistry. It requires specific antibodies directed against each of the proteins of interest (POI). However, the production of good quality primary antibodies is random and labor-intensive. Once obtained, it remains a resource with limited availability.

The advent of genetically targetable fluorescent protein tags has offered a possibility to bypass the requirement of antibody production against each POI. In addition, fluorescent tags have further expanded the field of possibilities to *in vivo* localization in living tissues or cells. Therefore, in *Drosophila*, where large scale projects are regularly conducted, various programs and consortiums have developed systematic approaches with the objective of creating lines expressing a fluorescent version of each protein of the proteome. Different approaches have been used to generate protein trap lines where an artificial exon encoding GFP is inserted into the genome (Morin et al., 2001; Clyne et al., 2004; Kelso et al., 2004; Buszczak et al., 2007; Quiñones-Coello et al., 2007; Lowe et al., 2014; Nagarkar-Jaiswal et al., 2015). Currently, the CRISPR technique better facilitates the creation of fusion proteins regulated by their endogenous environment, thereby the number of fluorescently tagged proteins generated by individual labs continuously increases.

In the vast majority of studies, the experiments described above are performed using light microscopes, which are fast, cheap and simple. The conventional fluorescence microscopy has a spatial resolution within a 200–300 nm range and it reaches a maximum of 10 nm in super-resolution microscopy but requires specialized equipment and/or fluorophores. In these conditions, intracellular localization often requires the co-localization with a fluorescent marker of organelles or compartments, although the size of many organelles is below the resolution limit of these microscopes.

Due to the imprecision of this approach, a high-resolution analysis becomes necessary through, for example, electron microscopy (EM)-based detection. Although EM achieves much higher spatial resolution (∼1 nm in biological samples), the localization of proteins by EM approaches remains rare. Several reasons lead to this situation. High quality results by EM immunolocalizations are difficult to obtain. Indeed, when performed on whole tissue, immunolocalization protocols include permeabilization steps that degrade intracellular structures. Alternatively, immunolocalizations performed on ultra-thin sections have only little epitope accessible to antibodies (Sosinsky et al., 2007; Schnell et al., 2012). Moreover, contrasting agents have a negative impact on the antigen-antibody binding, therefore protocols aim at maintaining them at low levels. This leads to images with poor contrast and makes the subsequent identification of ultrastructures difficult. Another alternative is then to perform ultrathin sections of cryoprotected samples infiltrated by 2.3 M sucrose followed by an immunolabeling of each section, which is both technically challenging and time consuming (Tokuyasu, 1986). Thus, there have been attempts to develop genetically encoded tags to circumvent these limitations, however, they either require light (mini-SOG) (Shu et al., 2011) or are not usable in most cellular compartments (HRP) (Porstmann et al., 1985). It is only the recent development of an engineered ascorbate peroxidase (APEX) that has allowed the use of tags in EM to be expanded (Martell et al., 2012). The APEX tag, derived from soybean ascorbate peroxidase (Lam et al., 2014), is a 28 kDa enzyme that converts the diffusible 3,3′-diaminobenzidine (DAB) into an insoluble osmiophilic polymer in the presence of H_2_O_2_. This polymer becomes EM-visible upon treatment by osmium tetroxide (OsO_4_). APEX has the advantage to retain activity after fixation with glutaraldehyde, a fixative that very well preserves the ultrastructure of the sample. APEX has been used as a tag in many studies and has largely proven its efficiency, making it now a tag of choice for the detection of fusion proteins in EM (Martell et al., 2012, 2017; Ariotti et al., 2015; Chen et al., 2015; Lee et al., 2016; Lin et al., 2016; Ludwig, 2020; Tan et al., 2020).

Here we have chosen to use the APEX2, a version of APEX (K14D, W41F, and E112K) with an additional mutation (A134P) (Lam et al., 2014). It has the same advantages of APEX while producing the DAB polymer with faster kinetics and incorporating the heme cofactor more efficiently. Similarly to an approach that has been reported to work successfully in zebrafish (Ariotti et al., 2015), we have created a GBP-APEX2 tool that combines the APEX2 with a GFP binding protein (GBP). The GBP corresponds to the coding sequence of an anti-GFP nanobody which is a single-domain polypeptide derived from the variable heavy chain (Vhh) of the heavy chain-only antibodies of camelids (Kirchhofer et al., 2010; Kubala et al., 2010). This GBP domain will allow the association of APEX with any GFP-tagged protein *in vivo*. In previous studies, it has been shown that individual intermediate filaments can be resolved (Ariotti et al., 2015) indicating that APEX-GBP allows a spatial resolution of ∼10 nm.

The APEX-GBP strategy avoids the need to create new APEX-tagged transgenic lines for each new POIs, and takes advantage of all the GFP-tagged lines already available in the *Drosophila* community. In addition, to further increase the adaptability and the versatility of our tool, we placed this fusion construct under the regulatory region of UAS sequences. These sequences trigger the expression of coding sequences placed downstream, when they are bound by the GAL4 transcription factor. Numerous *Drosophila* strains, with different expression patterns of GAL4 are available and thus, spatiotemporal control of expression can be achieved (Brand and Perrimon, 1993; Duffy, 2002).

**FIGURE 1:**
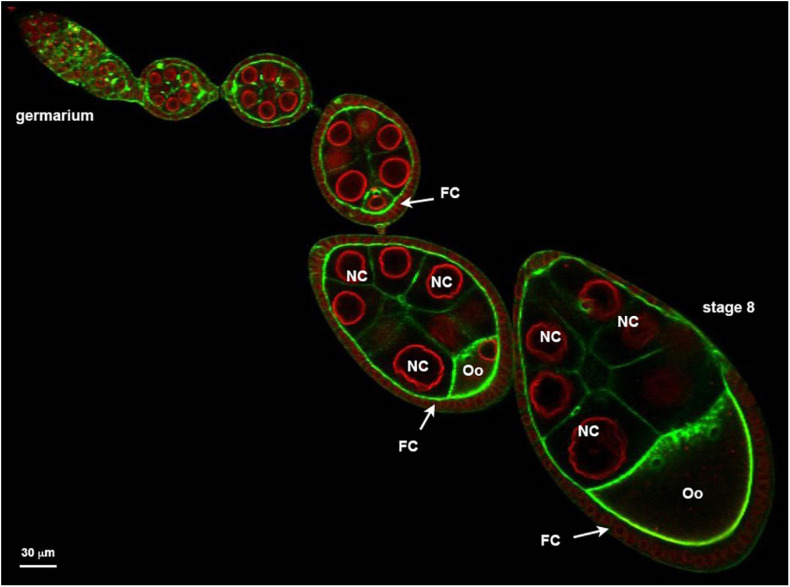
Ovariole organized in a succession of developing follicle. Nuclear envelopes (red) are stained with alexa594-WGA and the cortical actin (green) revealed with Alexa448-Phalloïdin. Follicles are developmental subunits in which the oocyte (Oo) develops. The oocyte is located at the posterior of the follicle and is associated with 15 additional germ cells named nurse cells (NC). A monolayer of follicular cells (FC) surrounds the germline. On the example presented here, the ovariole exhibits at its anterior extremity (left) a region called germarium in which the stem cells proliferate. At the other extremity (right) a stage 8 showing an oocyte that represents almost one half of the follicle.

As a proof of principle, we present here the localization of several GFP-tagged proteins and describe a detailed protocol applicable to the *Drosophila* ovarian follicle (Figure 1).

## Materials and Equipment

### Preliminary Notes

Many of the chemicals used during the APEX reaction and EM steps are hazardous to humans and the environment. Therefore, pay attention to the attached Material Safety Data Sheets and handle these reagents with care: glutaraldehyde (toxic), paraformaldehyde (carcinogen, toxic), DAB (carcinogen), cacodylate buffer (toxic, arsenic), hydrogen peroxide (corrosive), uranyl acetate (radioactive), lead citrate (toxic) and osmium tetroxide (highly volatile, toxic and highly reactive). Wearing gloves as well as adapted personal protective equipment and manipulating under a fume hood are essential.

### Fly Stocks

Fly strains and crosses were raised on standard cornmeal food at 25°C. To overexpress UAS transgenes specifically in germline cells, *nos-GAL4^*VP16*^* [*P(mw, GAL4:VP16-nos.UTR) CG6325*(*MVD1*)] (DGRC Kyoto #107955) and *mat-Tub-Gal4^*VP16*^* [*P(mw, mat-alpha4-Gal4-VP16)*] (Januschke et al., 2002) were used. To overexpress UAS transgenes in somatic follicular cells, *Tj-GAL4* [*y,w,; P(GawB)NP1624*) (DGRC Kyoto #104055] was used. The strains *UASp-APEX2-GBP* and *UASp-6myc-APEX2-GBP* are from this study. The following GFP strains were used: *P(Baz^*BAC.GFP*^)* aka *P[w* + , *FRT9-2]18E, f, baz [815.8], P{CaryP,PB[BAC Baz-sfGFP2]attP18}* (Besson et al., 2015); *P(mud^*BAC.GFP*^)* (Bosveld et al., 2016)*; UASp-Baz-GFP* (Benton and St Johnston, 2003); *UASp-Rab5-GFP* (Dong and Wu, 2013)*; hsp-flp; FRT79D ubi-nlsGFP (gift from JR Huynh); Tub-GFP-Rab6* (Januschke et al., 2007), *RanBP2-GFP* (Hampoelz et al., 2019).

### Generation of Transgenic Flies

APEX2-GBP from pCSDEST2 APEX2-GBP (Plasmid #67651, Addgene) was subcloned in pENTR^*TM*^/D-Topo. Using the Gateway^*TM*^ recombination cloning, APEX2-GBP sequence was inserted in pPMW (promotor UASp with a N-terminal 6myc tag), in pPW (promotor UASp without tag) from the Drosophila Gateway^*TM*^ Collection. The transgenic flies have been generated by random insertion by the BestGene Company (United States).

### Reagents and Equipment for Ovary Dissection and Immunostaining

• Bovine Serum Albumin (Thermo Fisher Scientific, BP1600).
• Chicken Anti-APEX2 antibody (Innovagen PA-APX2-100) raised against AA126-146.
• Mouse anti-Myc/c-Myc 9E10 antibody (SantaCruz, sc40).
• Anti-Mouse Secondary antibody, Alexa Fluor 546.
• Goat anti-Chicken IgY (H + L) Secondary Antibody, Alexa Fluor 546.
• Phosphate buffered saline pH 7.4 (PBS).
• Tween 20 (Sigma-Aldrich, P1379-1L).
• Triton X-100 (Sigma-Aldrich, T8787-100ML).
• Paraformaldehyde 16% (w/v) in sealed 10 mL glass ampules (Avantor, 43368.9L).
• Citifluor^*TM*^ Mountant Solution AF1 (Electron Microscopy Sciences, 17970-25).
• Forceps Dumont #5 (Carl Roth, K342.1).
• Stainless steel needles (Entosphinx, 20).
• Colorimetric 8 cell tray (Kartell Labware, 357).

### Reagents and Equipment for EM Sample Preparation and Detection

• Methylene blue staining solution (Richardson et al., 1960) (Methylene blue 0.5%, azur II 0.5%, Sodium borate 0.5%).
• Glass microscopy slides (Fisher Scientific, 1018049).
• Glutaraldehyde EM grade 25%, in sealed 10-ml glass ampules (EMS 16220).
• 3,3′-Diaminobenzidine tetrahydrochloride (DAB; Sigma-Aldrich, D5905).
• Hydrogen peroxide (H_2_O_2_), 3% (Boster Immunoleader AR1108).
• Sodium cacodylate buffer 0.2 M pH 7.4 (EMS 11652).
• Agar Low Viscosity resin Kit (Agar scientific, AGR1078).
• Fluoropolymer film 199 μm thickness (EMS, 50425).
• Formvar powder (Agar scientific AGR1202).
• Single slot grids (oval hole) (EMS, G2010-Cu).
• Osmium tetroxide 4%, in sealed 2-mL glass ampules (EMS, 19150).
• Potassium hexacyanoferrate(II) trihydrate (Sigma-Aldrich 244023).
• Ethanol.
• Uranyl acetate (AnalaaR 10288).
• Lead citrate (Deltamicroscopies 11300).

## Stepwise Procedure

### Fly Handling

In food vials, cross 5–10 virgin females with 3–5 males of the desired genotype and hold the vial at 25°C. After fly hatching select the females of the correct genotype, transfer them with few males in fresh food vials supplemented with dry yeast for their ovaries to fatten up and leave them for one or 2 days before dissection.

### Microdissection

1- Anesthetize the flies on a pad, with carbon dioxide.
2- Under the dissecting microscope, pick up one female with a pair of forceps and immerse it in a large drop (50–100 μL) of PBT (PBS with 0.1% Tween 20) at room temperature.
3- Hold the fly by the thorax with one pair of tweezers, and pull the dorsal abdominal cuticle around the A4–A5 segmental boundary with another pair of forceps.
4- Isolate and detach the pair of ovaries, which can fill up to 2/3 of the female abdomen, and should be readily available upon cuticle removal.
5- Tease apart the ovarioles of each ovary. While holding the posterior end of the ovaries (older stages) with a forceps, pass a needle in between the ovarioles toward the germarium at the anterior end of the ovary.
6- Transfer the ovaries into a 2 mL centrifuge tube containing 200 μL of PBT at room temperature and continue the experiment quickly.

### Fixation and DAB Reaction

7- Remove the PBT and add 500 μL of the fixative solution (2.5% paraformaldehyde, 1% glutaraldehyde in 0.1 M sodium cacodylate buffer).
8- Keep it 20 min at RT then move to 4°C.
9- Keep it at 4°C for 1 h, in the dark.
  *During this time, prepare the DAB solution*.
10- Wash three times 5 min in 0.1 M sodium cacodylate buffer at 4°C.
11- Prepare the DAB solution (1 mg/ml DAB, 0.1 M sodium cacodylate buffer). 1.5 mL per sample is required:
  - Dissolve one tablet of 10 mg DAB in 5 ml of H_2_O with 5 min vigorous vortexing.
  - Dilute 1:1 the DAB/H_2_O with 0.2 M sodium cacodylate buffer.
  - Remove undissolved precipitates with syringe filtration using a 0.2 μm filter (Millipore).
12- Add 500 μL of the final solution to the sample.
13- Allow to react for 30 min (*Increased time reduces background*).
14- Replace the solution with a DAB/Cacodylate + 5.88 mM H_2_O_2_ solution.
15- Incubate for 20 min at RT.
16- Stop the reaction with 3 min × 2 min washes with 0.1 M sodium cacodylate buffer.

### Post-Fixation

17- Prepare post-fixative solution.
  (a) 1% osmium tetroxide (prepared from 4% stock solution).
  (b) 1.5% Potassium hexacyanoferrate(II) trihydrate (prepared from stock powder).
  (c) 0.1 M cacodylate buffer (prepared from 0.2 M stock solution).
18- Incubate for 1 h at 4°C.
19- Wash three times 2 min in 0.1 M cacodylate buffer.
20- Wash three times 2 min in H_2_0.

### Dehydratation

21- Incubate 10 min in 30% EtOH solution.
22- Incubate 10 min in 50% EtOH solution.
23- Incubate 10 min in 70% EtOH solution.
24- Incubate 10 min in 90% EtOH solution.
25- Incubate twice 10 min in 100% EtOH solution.

### Resin

26- Incubate in resin LV agar/EtOH (1/1) overnight.
27- Incubate twice in resin for 1 h.
28- Mount the samples between two sheets of fluoropolymer film, separated by a fluoropolymer film spacer.
  This step is important because it allows the ovarioles to be laid out flat in order to select the right stage of development and to orientate them.
  28.1: Three pieces of fluoropolymer film embedding film are cut in the dimensions of a microscopy slide (75 mm × 25 mm).
  28.2: In the center of one of the three pieces of fluoropolymer film a square of 20 mm × 20 mm is cut out.
  28.3: Place the first piece of fluoropolymer film on a microscopy slide.
  28.4: Superimpose the hollowed film.
  28.5: Pipet the samples in resin.
  28.6: Spread out the ovarioles.
  28.7: Carefully apply the third piece of fluoropolymer film to minimizing the formation of bubbles.
  28.8: Put a microscopy slide on top to make a sandwich.
29- Leave the samples at 60°C for 18 h.
30- Select the stage of interest that will be subsequently processed using a light microscope.
31- Cut around the selected ovarian follicle and stick it flat on a block of resin with a drop of resin.
32- Leave the blocks at 60°C for 18 h.

### Cutting Sections and Contrasting

The samples are cut with an ultramicrotome. Select the area of interest in *z* by staining semi-thin sections (400 nm) with methylene blue solution. Then collect 70 nm ultra-thin sections on slot grids with an oval hole covered with formvar film. Classic grids with a square mesh can also be used depending on the cell model. In the case of the ovarian follicle, the object is too large and is partially hidden by the bars during the acquisition. Post-stain sections in 4% aqueous uranyl acetate in the dark for 15 min and lead citrate for 8 min in a CO_2_-depleted atmosphere created by the vicinity of sodium hydroxide tablets.

### Electron Microscopy Analysis

Observe the grids at 120 kV with a transmission electron microscope Tecnai12 (Thermo Fisher Scientific).

## Results

### Method Validation

When the method is performed for the first time or to troubleshoot the experiment, we recommend optimizing each step beforehand, including interaction between GBP-APEX2 and the GFP tagged POI, enzymatic activity of APEX2 and quality of the sample preparation.

#### Validating APEX2-GBP and GFP Expressions and Co-localization by Fluorescent Microscopy

We first verified that the GBP-APEX2 construction colocalized with the GFP tagged POI. This can be verified in the tissue using an immunofluorescence approach. Depending on the GBP-APEX2 strain used, immunostaining can be performed with the anti-APEX2 and/or anti-myc antibodies. Furthermore, this allows one to verify that the binding of APEX-GBP does not alter the localization of the GFP-tagged protein.

After microdissection (as described above), the ovaries should be treated according to the following protocol:

1- Fix with 4% paraformaldehyde (in PBS) for 12 min.
2- Wash twice 10 min with PBT2 (PBS + 0.1% Triton X-100).
3- Block in PBT2 + BSA 2% for 1 h.
4- Incubate in PBT2 + primary antibody overnight at 4°C. Anti-Myc antibody is used at 1/250 at anti-Apex2 at 1/500.
5- Wash three times 10 min with PBT2.
6- Incubate with secondary antibody in PBT2 for 2 h at room temperature.
7- Wash three times 10 min with PBT2.
8- Mount samples between slide and coverslip in a drop of citifluor^*TM*^.

**FIGURE 2:**
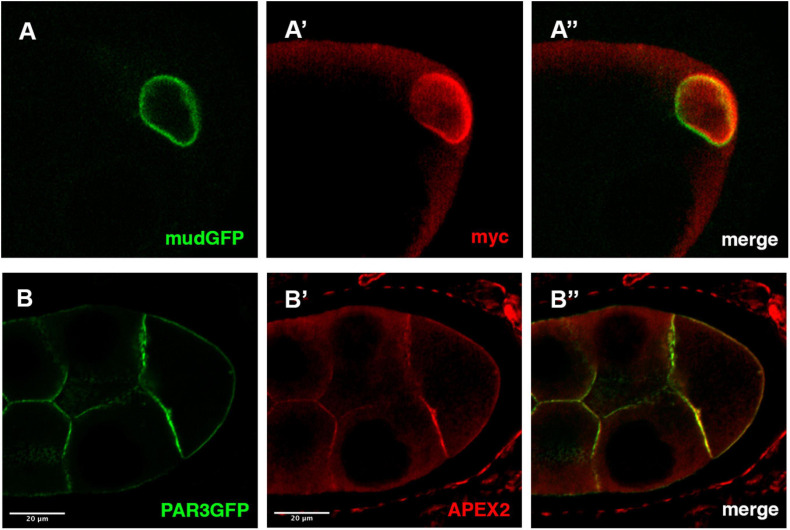
Detection APEX2-GFP protein by immunofluorescence. **(A,B)** In *mud-GFP*; *mat-Tub-Gal4^*VP16*^*, *UAS-mycAPEX2-GBP* ovarian follicle, the GFP signal is detected around the nucleus of the oocyte **(A)**. With anti-myc antibodies **(A’)**, we observe a similar pattern that overlaps with the GFP fluorescence **(A”)**. Similarly, in *nos-Gal4^*VP16*^*, *UAS-APEX2-GBP*, *UAS-Par3-GFP* egg chamber, we observe the GFP **(B)** associated with the membranes. Anti-APEX antibodies **(B’)** reveal an overlapping staining **(B”)**.

As a first example, we have performed immunofluorescence on ovaries expressing both a GFP version of the Mud/NuMA protein encoded by a BAC transgene and the UAS-myc-GBP-APEX2 construct expressed under the control of the mat-Tub-Gal4^VP16^ driver. Both GFP and anti-myc signals are colocalized at the nuclear envelope of the *Drosophila* oocyte (Figure 2A). This confirms that the GBP-APEX construct is able to correctly detect the GFP tagged protein. Similar results have been obtained when we undertook to detect the plasma membrane associated protein PAR3/Baz with the UAS-GBP-APEX construct and anti-APEX2 antibodies (Figure 2B).

It is noteworthy that the non-visualization of APEX signal at the exact localization of GFP fusion protein is not harmful for the rest of the experiment. Also, in some cases, a diffuse localization of the GBP-APEX can be detected in the cytoplasm without any consequence on subsequent precise detection of the GFP fusion protein (Supplementary Figure 1).

#### Validating APEX2 Activity by Light Microscopy

**FIGURE 3:**
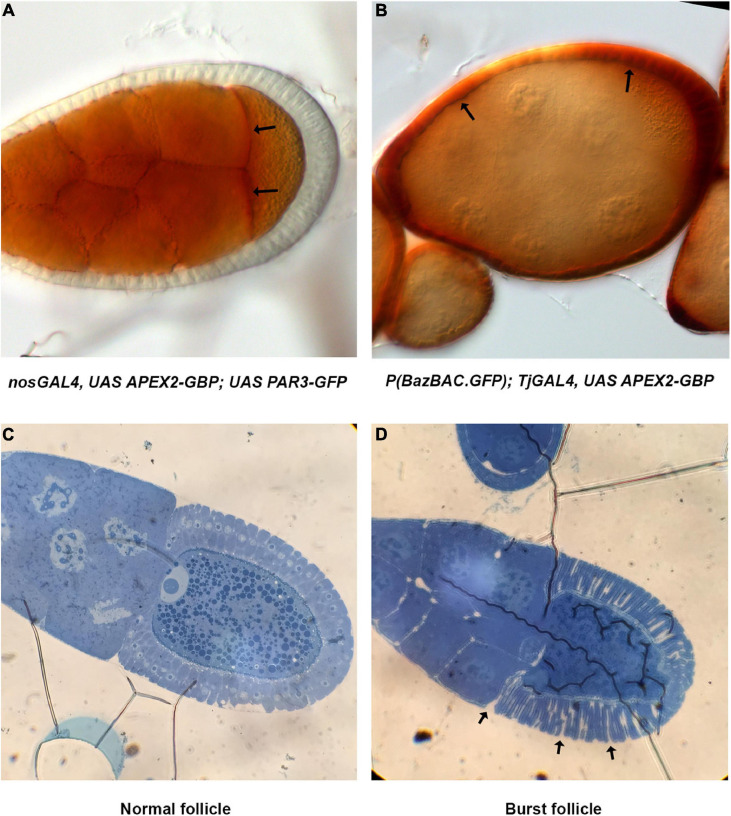
Method validation. **(A)** Images of *nos-Gal4^*VP16*^*, *UAS-APEX2-GBP*, *UAS-Par3-GFP* ovarian follicle acquired on transmission light microscope reveal DAB precipitates specifically in the germline. Stronger accumulation at the anterior of the oocyte (arrows) is coherent with Par3 distribution profile. **(B)** In *P(Baz^*BAC.GFP*^)*; *UAS-APEX2-GBP/Tj-GAL4*, DAB staining is only observed in follicular cells (arrows). Note that the brown shade over the germline staining is due to surrounding follicular cells. **(C,D)** Methylene blue staining reveals morphology of the follicle and allows visualization of nuclei. Thereby, we can also verify the developmental stage of the egg chamber **(C)**. In addition, issue with fixation is also revealed by burst ovarian follicles [arrows, **(D)**].

A critical step in this protocol is the ability of APEX2 to convert the DAB into a polymer. The polymer produced by the APEX enzymes is osmiophilic and thus can be visualized in EM, but it can also be visualized using light microscopy appearing as light brown stain (Figures 3A,B). Usually, the subcellular localization of APEX in the tissue can be roughly distinguished and bodes well for visualization in transmission EM (TEM). As an illustration, we decided to detect Baz-GFP with the GBP-APEX2 construct and look at the DAB product with a transmission light microscope. When the GBP-APEX2 is specifically expressed in the germline, under the regulatory sequences of nanos-GAL4 (*nos-GAL4^*VP16*^*), a brown precipitate is accumulated only in the nurse cells and the oocyte (Figure 3A). Moreover, we could clearly see a stronger accumulation at the anterior of the oocyte where PAR3/Baz is normally enriched. Alternatively, when the GBP-APEX2 is specifically driven in the follicular cells with the *traffic-jam-GAL4 (tj-GAL4)*, we observed a strong staining in the follicular epithelium that surrounds the ovarian follicle. The brown labeling is, as expected, more intense at the apical side of the cells (Figure 3B). The light brown staining observed over the germ cells corresponds to signals accumulated in the follicular cells above them. These experiments show that the fixation procedure does not alter the enzymatic activity of the APEX, nor the specificity of the DAB precipitate accumulation in cells expressing the GBP-APEX construct.

Of note the absence of accurate staining in transmission light microscopy does not indicate that no signal will be detected in TEM. However, if there is no staining at all, it is necessary to verify first that APEX is genetically present in the tissue/cell, and then that the labeling procedure with DAB is correctly performed.

#### Validating Sample Preparation

The quality of TEM preparation is a function of the correct completion of several crucial steps i.e., fixation, dehydration and embedding in a resin. The quality of embedding is very important and can be checked by the analysis of semi-thin sections of the sample under a light microscope. For thick samples like the *Drosophila* oocyte, this step can also be used to screen for adapted z-position before collecting ultra-thin sections for TEM. Semi-thin sections (0.4 μm thick) can be colored with methylene blue in order to better visualize cell morphology. Methylene blue staining allows one to recognize cell nuclei (Figure 3C) but also to reveal potential issues with the fixation step that could affect tissue morphology (Figure 3D).

After inclusion in resin (see above), we proceed to the following steps:

1- Semi-thin sections are generated with an ultramicrotome (Leica Microsystems UC6).
2- Two or three sections are deposited within a drop of water onto a glass slide.
3- Slides are placed 15–30 s on a preheated hot plate to dry the sections.
4- When the water is totally evaporated, apply a drop of the methylene blue staining solution onto the sections to cover the entire surface.
5- Incubate for 30 s on the hot plate.
6- Remove the staining solution by rinsing with distilled water.
7- Last traces of water are removed by placing the slide for 30 s back on the hot plate.
8- Observation under a dissecting microscope.

It should be noted that various polychromatic staining techniques can be used for embedded tissue sections (Toluidine blue, Basic fuchsin, and Malachite green).

### Proof of Principles

In order to validate our APEX tool in *Drosophila*, we have chosen to test, in the ovarian follicle, different GFP-tagged proteins associated with various subcellular compartments. These proteins are either overexpressed with the UAS/GAL4 system or expressed under the control of their own promoters with transgenes, or directly tagged in the genome by CRISPR mediated GFP insertion. For each condition, GFP was visualized by confocal microscopy, in parallel to the treatment for TEM observation. The APEX2-GBP construction is expressed under the control of UAS promoter and *nos-GAL4^*VP16*^* driver in the germline cells or *tj-GAL4* driver in the follicle cells.

**FIGURE 4:**
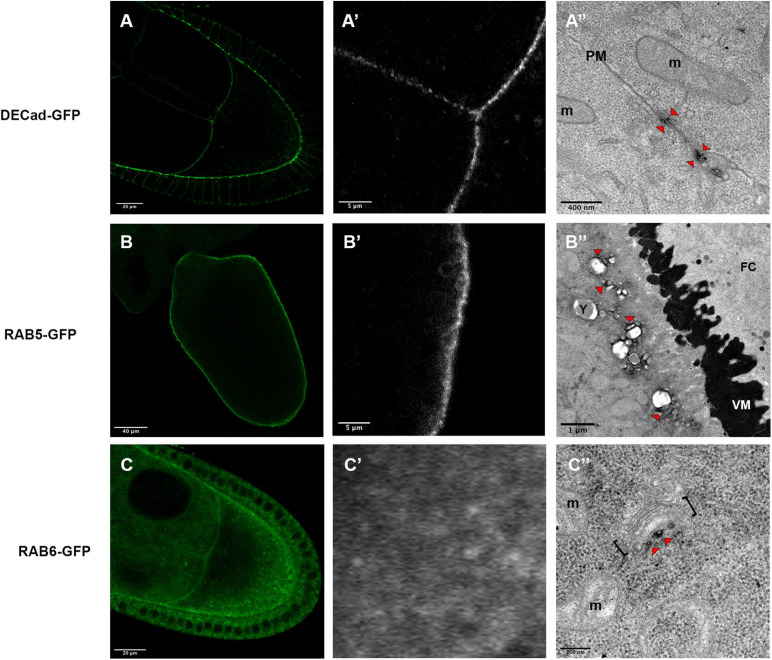
Expression profiles of *DE-Cad-GFP* **(A–A”)**, *Rab5-GFP* **(B–B”)** and *Rab6-GFP* **(C–C”)**, revealed by fluorescent microscopy **(A,A’,B,B’,C,C’)** or by electron microscopy **(A”,B”,C”)**. DE-Cad localizes at the plasma membrane [**(A)**, higher magnification **(A’)**]. DAB precipitates are visualized (arrowheads) near plasma membranes (PM) separating the oocyte and a nurse cell **(A”)**. Rab5 that has a cortical localization revealed by fluorescence [**(B)**, higher magnification **(B’)**], is detected at a small distance of the plasma membrane at the surfaces of vesicles [**(B”)**, arrowheads)]. Rab6-GFP displays a diffuse cytoplasmic signal **(C)** with some dots revealed at higher magnification **(C’)**. By electron microscopy, DAB precipitates are detected in the vicinity of Golgi apparatus [**(C”)**, arrowhead]. (m) mitochondria, brackets highlight a Golgi unit, (VM) vitelline membrane, (FC) follicular cells. Number of observations *DE-Cad-GFP* (*n* = 4), *Rab5-GFP* (*n* = 2) and *Rab6-GFP* (*n* = 6).

In a first attempt, we detected DE-Cadherin (DE-Cad) encoded by the *shotgun* gene in *Drosophila.* DE-Cad is a component of the adherens junction that localizes at the plasma membrane. Using a DE-Cad-GFP knockin line (Huang et al., 2009), we observed by fluorescence a signal at the oocyte plasma membrane with patches of higher intensity (Figures 4A,A’). Similarly, APEX detection and visualization by TEM revealed electron dense patches associated with plasma membranes (Figure 4A”).

We then addressed whether this approach is suitable to detect protein involved in cellular trafficking, such as the GTPases RAB5 and RAB6. Fluorescence detection of the RAB5-GFP tagged protein, expressed under the control of UAS sequence, displays a cortical signal along the plasma membrane of the oocyte (Figures 4B,B’) as expected with the previously described RAB5 association with early endosomes (Zerial and McBride, 2001; Compagnon et al., 2009). With our detection method by TEM, we have observed signals associated with vesicles near the plasma membrane (Figure 4B”). Interestingly the DAB precipitate seems to be organized in nanodomains on the endosomes as it has been proposed previously (Franke et al., 2019). Concerning RAB6, this GTPase is known to be associated with medial Golgi and *Trans* Golgi Network (Antony et al., 1992) and it has been shown to regulate transport between early and late Golgi compartments and to sustain Golgi morphology (White et al., 1999; Januschke et al., 2007). With fluorescence we observed a diffuse staining pattern, with a few more intense dots, scattered within the cytoplasm (Figures 4C,C’). APEX-GBP revealed by TEM highly contrasted dots in the close vicinity of Golgi apparatus, a location consistent with the previously described role of RAB6 (Figure 4C”).

**FIGURE 5:**
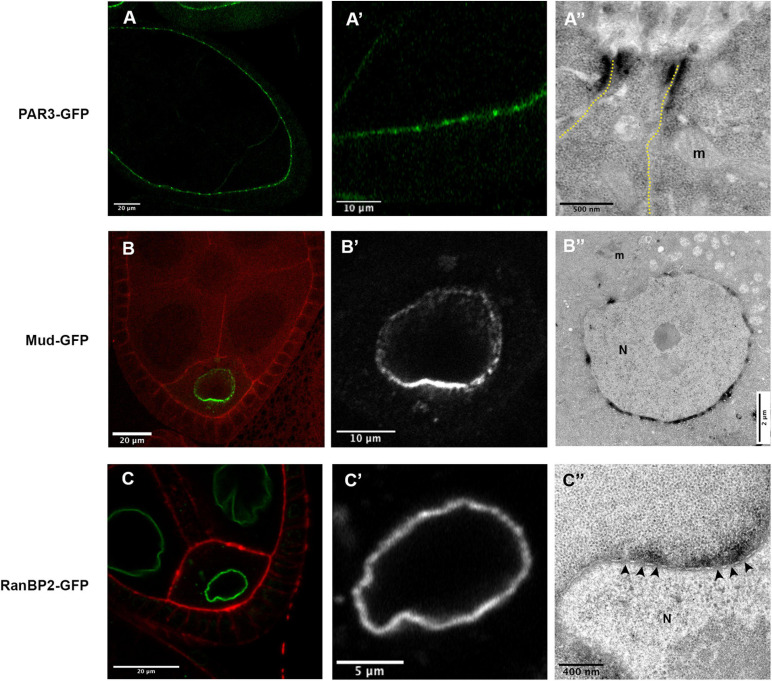
**(A)** In P(Baz^BAC.GFP^); *Tj-GAL4*, *UAS-APEX2-GBP* egg chambers, Baz-GFP accumulate apically in the follicular cells, with a stronger accumulation at the junctional level **(A,A’)**. Consistently, a dense signal is revealed at the level of the junction between two follicular cells **(A”)**. **(B)** In *mud*^4^/*mud*^4^; *P(mud^*BAC.GFP*^)*/*P(mud^*BAC.GFP*^)*; *nos-GAL4^*VP16*^*, *UAS-APEX2-GBP* follicles, an asymmetric distribution of Mud-GFP is observed at the nuclear envelope of the oocyte **(B,B’)**. This accumulation, more important on the hemisphere facing the posterior membrane of the oocyte, is also revealed by the APEX-GBP tool by electron microscopy. **(C)** In *RanBP2-GFP*; *nos-GAL4^*VP16*^*, *UAS-APEX2-GBP* egg chamber the GFP signal is detected at the nuclear envelope [**(C’)**, zoom on oocyte nucleus (N) in panel **(C”)**]. By electron microscopy, we can observe that the accumulation of DAB precipitate is outside the nucleus and correlates with the presence of nuclear pores (arrowheads). (N) nucleus, (m) mitochondria. Number of observations **(A)**: (*n* = 5), **(B,C)** (*n* = 2).

To monitor the versatility of the UAS-GBP-APEX2 tool, we tested the use of this tool in the somatic cells surrounding the ovarian follicle. For this purpose, we monitored the localization of the PAR3/Baz polarity protein tagged with GFP and expressed at endogenous levels. Upon APEX detection and visualization by TEM, dense patches were easily identified at the level of the adherens junctions as expected for PAR3/Baz (Figures 5A,A’ and Supplementary Figure 1). The APEX-GBP system is therefore effective in tracking the EM localization of GFP-tagged proteins independently of their expression level.

To test, if the method is sensitive enough to reflect differences in protein quantity, we chose to follow the nuclear envelope repartition of the Mud/NuMA protein, that is known to be asymmetrically distributed at the oocyte nuclear envelope (Yu et al., 2006; Tissot et al., 2017; Figures 5B,B’). Accordingly, the ultra-thin sections observed in TEM exhibit more dense signals on the portion of the nuclear envelope facing the posterior membrane of the oocyte (Figure 5B”). DAB precipitates on the opposite side of the nucleus are much less present showing that our conditions can detect different quantities of proteins.

One limit of the APEX approach is the diffusion of the DAB precipitate formed by the enzymatic activity. We thus decided to estimate this diffusion by looking at a protein with a precise location, i.e., the RanBP2/Nup358 protein that is an outer component of nuclear pore complexes (Bernad et al., 2004). Strikingly, the diffusion observed with the detection of RanBP2-GFP is restricted outside of the nucleus according to the known location of the protein (Figure 5C). Furthermore, we do not detect staining when large portions of NE devoid of visible nuclear pores are observed. In this case, no diffusion could be detected inside the nucleus, showing that this method is suitable to decipher if a given protein is associated with the inner or outer membrane of the NE.

**FIGURE 6:**
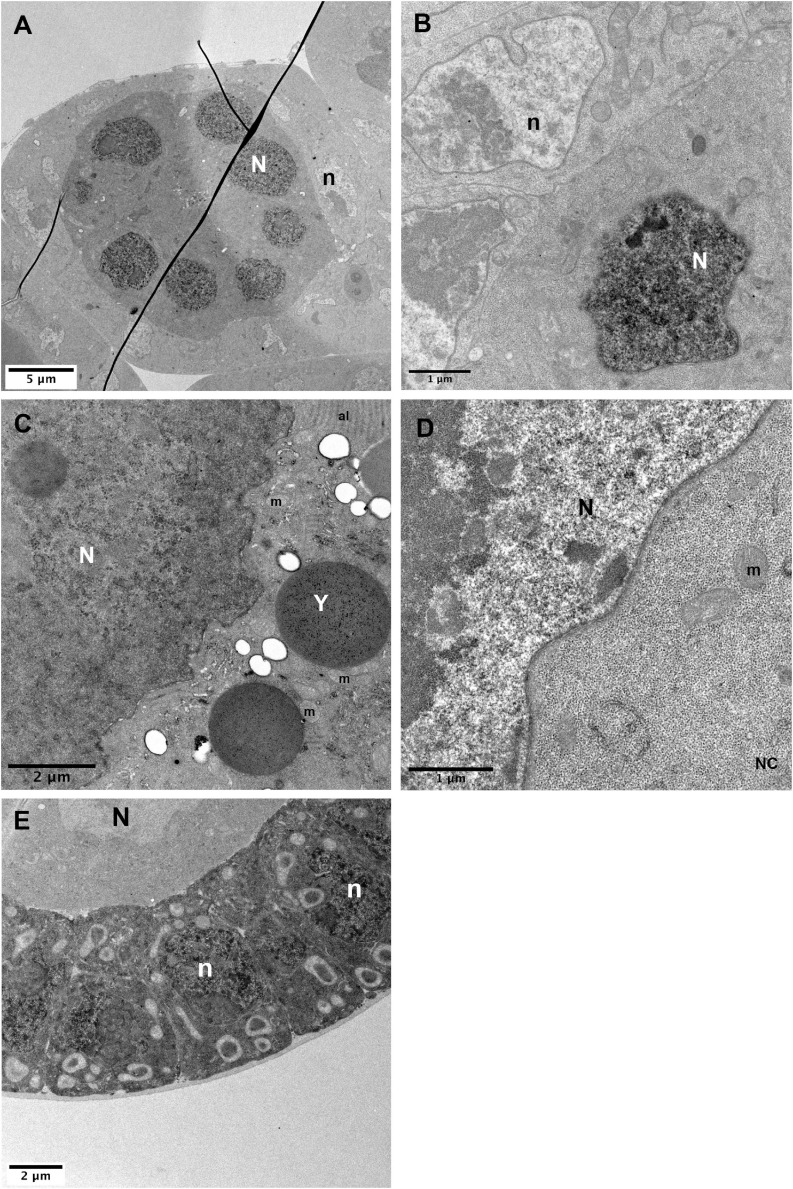
Nuclear detection of APEX2. **(A–C)** In *hsp-flp*; *FRT79D ubi-nlsGFP/nos-GAL4^*VP16*^*, *UAS-APEX2-GBP* follicles, strong DAB staining is observed in germline nuclei (N) [**(A)**, higher magnifications in panels **(B,C)**]. **(D)** Control sample without the APEX2-GBP transgene exhibits a strong difference between the contrasts of the nucleus that is lighter than the cytoplasm. Comparison of the contrasts between panels **(C,D)** clearly shows that the stronger signal in the nucleus of *hsp-flp*; *FRT79D ubi-nlsGFP/nos-GAL4^*VP16*^*, *UAS-APEX2-GBP* ovarian follicles is specific. **(E)** In *hsp-flp*; *Tj-GAL4*; *FRT79D ubi-nlsGFP/UAS-APEX2-GBP*, nuclei of the follicular cells display strong accumulation of DAB precipitates. (N) germline nuclei, (n) follicle cells nuclei, (Y) yolk vesicle, (m) mitochondria, (al) annulate lamellae. Number of observations **(A–C)**: (*n* = 5), **(D)** (*n* = 2), **(E)** (*n* = 2).

This observation prompted us to test if the UAS-GBP-APEX2 tool could be used to detect protein within the nucleus, despite our choice not to include a nuclear localization sequence in our GBP-APEX construct. In order to address if a nucleus-resident protein could nevertheless be monitored in TEM using our APEX-GBP tool, we expressed a nls-GFP transgene concomitantly with APEX-GBP in the ovarian follicle (Figure 6). In Figure 6A, the APEX-GBP is expressed only in the germline and we can observe a more intense staining in the germline nuclei (Figure 6, white N). Note that a weak staining in the germline cytoplasm can also be observed indicating the presence of APEX-GBP not associated with GFP. Importantly, in the follicle cells that serve as a control condition, the nucleus is lighter than the surrounding cytoplasm (Figures 6A,B, black n), unlike the germline where the nucleus is more strongly stained (Figures 6A,B, white N) than the cytoplasm.

## Discussion: Critical Parameters and Troubleshooting

### Driver/APEX-GBP Couple

Having a bi-partite system where the APEX is uncoupled from the POI has many advantages as mentioned previously. However, in this system APEX is expressed throughout the whole cell independently of the POI’s subcellular location and thereby induces a background signal. Therefore, it is important to perform control experiments when studying a protein for the first time (see below). This is also exemplified by the detection of signals unspecific to our protein when immunofluorescence experiments are performed with anti-APEX antibodies (see Figure 2). However, in TEM, this does not prevent an accurate detection of Mud only at the nuclear envelope of the oocyte. It is noteworthy that negative controls display much lower global signal, indicating that a significant but acceptable level of noise is induced by this condition. We can speculate that the local concentration of APEX protein is higher when bound to the POI and thereby create a signal/noise ratio in favor of the detection.

When we used the *Tj-GAL4* driver to detect the nls-GFP in the follicular cells, we observed a cytoplasmic background of a similar level to the nuclear signal associated with the nls-GFP transgene (Figure 6E). It is then difficult without control to distinguish between specific and non-specific labeling. Nonetheless, in the same cells and with the same driver, the signal of PAR3/Baz-GFP, expressed at endogenous levels with a BAC transgene, was strong enough to be unambiguously identified (Figure 5A”). These examples reveal that the driver/APEX-GBP couple has to be carefully chosen in order to maximize the signal to noise ratio. It is noteworthy that several methods have recently been reported to improve the signal-noise ratio, i.e., the use of conditionally stable nanobodies for GFP fused to APEX, that favor degradation of unbound nanobodies by the proteasome reduces background APEX signals (Ariotti et al., 2018), or the possibility to convert the oxidized diaminobenzidine reaction product of APEX into a silver/gold particle that in addition provides a readily quantifiable particulate signal (Rae et al., 2021). Both approaches have been proven successful in cell cultures and remain to be tested and adapted *in vivo* to thicker tissue like Drosophila follicles.

### Negative Controls

Transmission EM images are displayed in gray levels that reflect the density of the structure encountered by the electron beam. In order to help visualize cellular structures and increase the contrast, the samples must be incubated with osmium tetroxide, uranyl acetate and/or potassium ferricyanide. DAB precipitates also appear as dense structures, thereby it could be challenging to identify the osmiophilic precipitate produced by APEX especially when the POI has an unknown location. Therefore, we suggest performing negative controls with samples devoid of APEX-GBP proteins (no GAL4 transgene or no UAS-APEX2-GBP transgenes) and samples lacking GFP proteins. If a staining is reproducibly observed in APEX expressing tissue and never observed in controls, we can be confident about the specificity of the staining. In the case of nls-GFP localization with APEX, we thus compared the contrasts existing in the oocyte nucleus in the presence (Figure 6C) or absence (Figure 6D) of the APEX2 transgene. We also observed APEX-related contrast in the absence of GFP-labeled proteins (Supplementary Figure 2).

### Golgi Apparatus

In the Drosophila ovarian follicle, we often visualized an electron dense staining, independently of the presence of APEX, within the Golgi cisternae (Supplementary Figure 2). We believe that it depends on a glutathione peroxidase (PHGPx) located in the Golgi apparatus (Missirlis et al., 2003). The visualization of this staining is not fully penetrant but the APEX-GBP tool is probably not appropriate for the detection of POI located in this organelle.

### Weak APEX Signal

H_2_O_2_ is necessary for the oxidation reaction to occur, however, it has also been reported that long incubation could inhibit the reaction (Ludwig, 2020). Therefore, in case of weak signal, the DAB labeling can not very easily be adjusted by changing its duration. Instead it has been suggested that lowering H_2_O_2_ concentration to 0.5 mM greatly enhances APEX2 activity and sensitivity, and results in an increased contrast in TEM (Ludwig, 2020). It is therefore possible that small amounts of APEX protein can be detected by adjusting the H_2_O_2_ concentration within the 10–0.5 mM range. Here we used in routine an intermediate concentration of 5.9 mM.

### Time Considerations

Dissection, fixation and washing procedures take around 4 h and are followed by 1 h post-fixation incubation, 1 h dehydratation before an overnight incubation in the resin. Embedding takes a further 24 h, followed by an additional day required for resin polymerization. The embedded sample can be stored indefinitely before sectioning.

## Conclusion

The use of APEX has recently gained momentum in the scientific community as it offers an easy, cheap and rapid way of localizing a POI with the resolution of EM. This peroxidase has already been used in *Drosophila* in its original version (Chen et al., 2015; Lin et al., 2016).

By coupling APEX2 to the GBP nanobody, we have created a new tool that can be used in any cell type and for any GFP (and derivatives) labeled protein in Drosophila. We show here the flexibility of this tool to identify the nanometric localization of proteins in different compartments by TEM. Notably, proteins either expressed by their endogenous promoters or over-expressed have been detected with the exact same conditions, showing that this protocol does not need too much adaptation from one POI to another. We have tested our protocol with classical TEM, but there have been reports showing that EM volume imaging such as SBF (serial block face) technology could also be successfully combined with APEX approaches (data not shown and Ariotti et al., 2015; Ludwig, 2020).

The bi-partite detection of APEX also offers the possibility for a reliable technique of correlative light and EM (CLEM) whereby the GFP-tagged POI can be visualized using fluorescence microscopy, and the DAB precipitate generated by APEX can be identified by EM at the place of the GFP-tag (For review, see Ariotti et al., 2015; Ludwig, 2020).

Finally, another popular use of APEX are the proteomic approaches. Indeed, in addition to DAB, APEX can use biotin-phenol as substrate. In presence of H_2_O_2_, APEX then catalyzes the formation of biotin-phenoxyl, which can covalently bind electron-rich amino acids such as tyrosine in the proteins located in close proximity. It is estimated that modified proteins are within a radius of 20 nm. Biotinylated proteins are subsequently identified by mass spectrometry. Several studies have successfully developed this approach including in *Drosophila* (Markmiller et al., 2018; Marmor-Kollet et al., 2020) and the *Drosophila* oocyte (Mannix et al., 2019; Gerdes et al., 2020). In addition, as APEX can also biotinylate guanosine in RNA, a recent study has used this property to determine subcellular transcriptome after RNA sequencing (Fazal et al., 2019). All these studies have been performed by using direct fusion of APEX to a POI. Theoretically, our APEX-GBP tool could also be suitable for these approaches and would prevent labs from generating new constructs given all the already existing GFP-tagged proteins.

## Data Availability Statement

The original contributions presented in the study are included in the article/Supplementary Material, further inquiries can be directed to the corresponding author/s.

## Author Contributions

JJ and SC designed the project and performed the clonage and transgenic analysis. SC and FB performed *Drosophila* experiments. CD and RB performed the TEM experiments. SC and FB conducted data interpretation and writing. All authors contributed to manuscript revision, read, and approved the submitted version.

## Conflict of Interest

The authors declare that the research was conducted in the absence of any commercial or financial relationships that could be construed as a potential conflict of interest.

## Publisher’s Note

All claims expressed in this article are solely those of the authors and do not necessarily represent those of their affiliated organizations, or those of the publisher, the editors and the reviewers. Any product that may be evaluated in this article, or claim that may be made by its manufacturer, is not guaranteed or endorsed by the publisher.

## Supplementary Material

Supplementary Figure 1 Immunostaining anti-APEX2 on *P(Baz^*BAC.GFP*^)*; *Tj-GAL4*, *UAS-APEX2-GBP* ovarian follicles. Specific but diffuse staining is revealed in follicular cells.

Supplementary Figure 2 **(A)** Examples of non-specific staining independent of APEX in Golgi apparatus. **(B)** Negative control of APEX experiment. The APEX procedure was realized on *mud*^4^/*mud*^4^; *P(mud^*BAC.GFP*^)/P(mud^*BAC.GFP*^*). In absence of APEX2-GBP, we revealed only the cell structures. (G) Golgi apparatus, (N) nuclei, (m) mitochondria, (PM) plasma membrane, (ER) endoplasmic reticulum.

## References

[B1] Antony C. Cibert C. Geraud G. Santa Maria A. Maro B. Mayau V. (1992). The small GTP-binding protein rab6p is distributed from medial Golgi to the trans-Golgi network as determined by a confocal microscopic approach. *J. Cell Sci.* 103 785–796. 10.1242/jcs.103.3.785 1478971

[B2] Ariotti N. Hall T. E. Rae J. Ferguson C. McMahon K. A. Martel N. (2015). Modular detection of GFP-labeled proteins for rapid screening by electron microscopy in cells and organisms. *Dev. Cell* 35 513–525. 10.1016/j.devcel.2015.10.016 26585296

[B3] Ariotti N. Rae J. Giles N. Martel N. Sierecki E. Gambin Y. (2018). Ultrastructural localisation of protein interactions using conditionally stable nanobodies. *PLoS Biol.* 16:e2005473. 10.1371/journal.pbio.2005473 29621251 PMC5903671

[B4] Benton R. St Johnston D. (2003). Drosophila PAR-1 and 14-3-3 inhibit Bazooka/PAR-3 to establish complementary cortical domains in polarized cells. *Cell* 115 691–704. 10.1016/s0092-8674(03)00938-3 14675534

[B5] Bernad R. van der Velde H. Fornerod M. Pickersgill H. (2004). Nup358/RanBP2 attaches to the nuclear pore complex via association with Nup88 and Nup214/CAN and plays a supporting role in CRM1-mediated nuclear protein export. *Mol. Cell. Biol.* 24 2373–2384. 10.1128/mcb.24.6.2373-2384.2004 14993277 PMC355853

[B6] Besson C. Bernard F. Corson F. Rouault H. Reynaud E. Keder A. (2015). Planar cell polarity breaks the symmetry of PAR protein distribution prior to mitosis in *Drosophila* sensory organ precursor cells. *Curr. Biol.* 25 1104–1110. 10.1016/j.cub.2015.02.073 25843034

[B7] Bosveld F. Markova O. Guirao B. Martin C. Wang Z. Pierre A. (2016). Epithelial tricellular junctions act as interphase cell shape sensors to orient mitosis. *Nature* 530 495–498. 10.1038/nature16970 26886796 PMC5450930

[B8] Brand A. H. Perrimon N. (1993). Targeted gene expression as a means of altering cell fates and generating dominant phenotypes. *Development* 118 401–415. 10.1242/dev.118.2.401 8223268

[B9] Buszczak M. Paterno S. Lighthouse D. Bachman J. Planck J. Owen S. (2007). The carnegie protein trap library: a versatile tool for drosophila developmental studies. *Genetics* 175 1505–1531. 10.1534/genetics.106.065961 17194782 PMC1840051

[B10] Chen C. L. Hu Y. Udeshi N. D. Lau T. Y. Wirtz-Peitz F. He L. (2015). Proteomic mapping in live *Drosophila* tissues using an engineered ascorbate peroxidase. *Proc. Natl. Acad. Sci. U.S.A.* 112 12093–12098. 10.1073/pnas.1515623112 26362788 PMC4593093

[B11] Clyne P. J. Brotman J. S. Sweeney S. T. Davis G. (2004). Erratum: green fluorescent protein tagging drosophila proteins at their native genomic loci with small P elements. *Genetics* 167:2143. 10.1093/genetics/167.4.2763a PMC1462835 14668392

[B12] Compagnon J. Gervais L. San Roman M. Chamot-Bœuf S. Guichet A. (2009). Interplay between Rab5 and PtdIns(4,5)P2 controls early endocytosis in the *Drosophila* germline. *J. Cell Sci.* 122 25–35. 10.1242/jcs.033027 19050045

[B13] Dong C. Wu G. (2013). G-protein-coupled receptor interaction with small GTPases. *Methods Enzymol.* 522 97–108. 10.1016/b978-0-12-407865-9.00006-6 23374182 PMC3608118

[B14] Duffy J. B. (2002). GAL4 system in *Drosophila*: a fly geneticist’s Swiss army knife. *Genesis* 34 1–15. 10.1002/gene.10150 12324939

[B15] Fazal F. M. Han S. Parker K. R. Kaewsapsak P. Xu J. Boettiger A. N. (2019). Atlas of Subcellular RNA localization revealed by APEX-Seq. *Cell* 178 473–490. 10.1016/j.cell.2019.05.027 31230715 PMC6786773

[B16] Franke C. Repnik U. Segeletz S. Brouilly N. Kalaidzidis Y. Verbavatz J. M. (2019). Correlative single-molecule localization microscopy and electron tomography reveals endosome nanoscale domains. *Traffic* 20 601–617. 10.1111/tra.12671 31206952 PMC6771687

[B17] Gerdes J. A. Mannix K. M. Hudson A. M. Cooley L. (2020). HtsRc-mediated accumulation of f-actin regulates ring canal size during drosophila melanogaster oogenesis. *Genetics* 216 717–734. 10.1534/genetics.120.303629 32883702 PMC7648574

[B18] Hampoelz B. Schwarz A. Ronchi P. Bragulat-Teixidor H. Tischer C. Gaspar I. (2019). Nuclear pores assemble from nucleoporin condensates during oogenesis. *Cell* 179 671–686. 10.1016/j.cell.2019.09.022 31626769 PMC6838685

[B19] Huang J. Zhou W. Dong W. Watson A. M. Hong Y. (2009). Directed, efficient, and versatile modifications of the *Drosophila* genome by genomic engineering. *Proc. Natl. Acad. Sci. U.S.A.* 106 8284–8289. 10.1073/pnas.0900641106 19429710 PMC2688891

[B20] Januschke J. Gervais L. Dass S. Kaltschmidt J. A. Lopez-Schier H. St Johnston D. (2002). Polar transport in the *Drosophila* oocyte requires dynein and kinesin I cooperation. *Curr. Biol.* 12 1971–1981. 10.1016/s0960-9822(02)01302-7 12477385

[B21] Januschke J. Nicolas E. Compagnon J. Formstecher E. Goud B. Guichet A. (2007). Rab6 and the secretory pathway affect oocyte polarity in *Drosophila*. *Development* 134 3419–3425. 10.1242/dev.008078 17827179

[B22] Kelso R. J. Buszczak M. Quiñones A. T. Castiblanco C. Mazzalupo S. Cooley L. (2004). Flytrap, a database documenting a GFP protein-trap insertion screen in *Drosophila melanogaster*. *Nucleic Acids Res.* 32 D418–D420. 10.1093/nar/gkh014 14681446 PMC308749

[B23] Kirchhofer A. Helma J. Schmidthals K. Frauer C. Cui S. Karcher A. (2010). Modulation of protein properties in living cells using nanobodies. *Nat. Struct. Mol. Biol.* 17 133–139. 10.1038/nsmb.1727 20010839

[B24] Kubala M. H. Kovtun O. Alexandrov K. Collins B. M. (2010). Structural and thermodynamic analysis of the GFP:GFP-nanobody complex. *Protein Sci.* 19 2389–2401. 10.1002/pro.519 20945358 PMC3009406

[B25] Lam S. S. Martell J. D. Kamer K. J. Deerinck T. J. Ellisman M. H. Mootha V. K. (2014). Directed evolution of APEX2 for electron microscopy and proximity labeling. *Nat. Methods* 12 51–54. 10.1038/nmeth.3179 25419960 PMC4296904

[B26] Lee S.-Y. Kang M.-G. Park J.-S. Lee G. Ting A. Y. Rhee H.-W. (2016). APEX Fingerprinting reveals the subcellular localization of proteins of interest. *Cell Rep.* 15 1837–1847. 10.1016/j.celrep.2016.04.064 27184847

[B27] Lin T. Y. Luo J. Shinomiya K. Ting C. Y. Lu Z. Meinertzhagen I. A. (2016). Mapping chromatic pathways in the *Drosophila* visual system. *J. Comp. Neurol.* 524 213–227. 10.1002/cne.23857 26179639 PMC4678965

[B28] Lowe N. Rees J. S. Roote J. Ryder E. Armean I. M. Johnson G. (2014). Analysis of the expression patterns, subcellular localisations and interaction partners of drosophila proteins using a pigp protein trap library. *Development* 141 3994–4005. 10.1242/dev.111054 25294943 PMC4197710

[B29] Ludwig A. (2020). “Selective visualization of caveolae by tem using apex2,” in *Methods in Molecular Biology*, ed. Blouin C. (New York, NY: Humana Press Inc), 1–10. 10.1007/978-1-0716-0732-9_1 32548814

[B30] Mannix K. M. Starble R. M. Kaufman R. S. Cooley L. (2019). Proximity labeling reveals novel interactomes in live Drosophila tissue. *Development* 146:dev176644. 10.1242/dev.176644 31208963 PMC6679357

[B31] Markmiller S. Soltanieh S. Server K. L. Mak R. Jin W. Fang M. Y. (2018). Context-dependent and disease-specific diversity in protein interactions within stress granules. *Cell* 172 590–604. 10.1016/j.cell.2017.12.032 29373831 PMC5969999

[B32] Marmor-Kollet H. Siany A. Kedersha N. Knafo N. Rivkin N. Danino Y. M. (2020). Spatiotemporal proteomic analysis of stress granule disassembly using APEX reveals regulation by SUMOylation and links to ALS pathogenesis. *Mol. Cell* 80 876–891. 10.1016/j.molcel.2020.10.032 33217318 PMC7816607

[B33] Martell J. D. Deerinck T. J. Lam S. S. Ellisman M. H. Ting A. Y. (2017). Electron microscopy using the genetically encoded APEX2 tag in cultured mammalian cells. *Nat. Protoc.* 12 1792–1816. 10.1038/nprot.2017.065 28796234 PMC5851282

[B34] Martell J. D. Deerinck T. J. Sancak Y. Poulos T. L. Mootha V. K. Sosinsky G. E. (2012). Engineered ascorbate peroxidase as a genetically encoded reporter for electron microscopy. *Nat. Biotechnol.* 30 1143–1148. 10.1038/nbt.2375 23086203 PMC3699407

[B35] Missirlis F. Rahlfs S. Dimopoulos N. Bauer H. Becker K. Hilliker A. (2003). A putative gluatathione peroxidase of Drosophila encodes a thioredoxin peroxidase that provides resistance against oxidative stress but fails to complement a lack of catalase activity. *Biol. Chem.* 384 463–472. 10.1515/BC.2003.052 12715897

[B36] Morin X. Daneman R. Zavortink M. Chia W. (2001). A protein trap strategy to detect GFP-tagged proteins expressed from their endogenous loci in *Drosophila*. *Proc. Natl. Acad. Sci. U.S.A.* 98 15050–15055. 10.1073/pnas.261408198 11742088 PMC64981

[B37] Nagarkar-Jaiswal S. Lee P. T. Campbell M. E. Chen K. Anguiano-Zarate S. Gutierrez M. C. (2015). A library of MiMICs allows tagging of genes and reversible, spatial and temporal knockdown of proteins in *Drosophila*. *Elife* 4:e05338. 10.7554/eLife.05338 25824290 PMC4379497

[B38] Porstmann B. Porstmann T. Nugel E. Evers U. (1985). Which of the commonly used marker enzymes gives the best results in colorimetric and fluorimetric enzyme immunoassays: horseradish peroxidase, alkaline phosphatase or β-galactosidase? *J. Immunol. Methods* 79 27–37. 10.1016/0022-1759(85)90388-6 3923120

[B39] Quiñones-Coello A. T. Petrella L. N. Ayers K. Melillo A. Mazzalupo S. Hudson A. M. (2007). Exploring strategies for protein trapping in drosophila. *Genetics* 175 1089–1104. 10.1534/genetics.106.065995 17179094 PMC1840052

[B40] Rae J. Ferguson C. Ariotti N. Webb R. I. Cheng H. H. Mead J. L. (2021). A robust method for particulate detection of a genetic tag for 3D electron microscopy. *Elife* 10:e64630. 10.7554/ELIFE.64630 33904409 PMC8104959

[B41] Richardson K. C. Jarett L. Finke E. H. (1960). Embedding in epoxy resins for ultrathin sectioning in electron microscopy. *Biotech. Histochem.* 35 313–323. 10.3109/10520296009114754 13741297

[B42] Schnell U. Dijk F. Sjollema K. A. Giepmans B. N. G. (2012). Immunolabeling artifacts and the need for live-cell imaging. *Nat. Methods* 9 152–158. 10.1038/nmeth.1855 22290187

[B43] Shu X. Lev-Ram V. Deerinck T. J. Qi Y. Ramko E. B. Davidson M. W. (2011). A genetically encoded tag for correlated light and electron microscopy of intact cells, tissues, and organisms. *PLoS Biol.* 9:1001041. 10.1371/journal.pbio.1001041 21483721 PMC3071375

[B44] Sosinsky G. E. Giepmans B. N. G. Deerinck T. J. Gaietta G. M. Ellisman M. H. (2007). Markers for correlated light and electron microscopy. *Methods Cell Biol.* 2007 575–591. 10.1016/S0091-679X(06)79023-9 17327175

[B45] Tan B. Yatim S. M. J. M. Peng S. Gunaratne J. Hunziker W. Ludwig A. (2020). The mammalian crumbs complex defines a distinct polarity domain apical of epithelial tight junctions. *Curr. Biol.* 30 2791–2804. 10.1016/j.cub.2020.05.032 32531288

[B46] Tissot N. Lepesant J. A. Bernard F. Legent K. Bosveld F. Martin C. (2017). Distinct molecular cues ensure a robust microtubule-dependent nuclear positioning in the *Drosophila* oocyte. *Nat. Commun.* 8:15168. 10.1038/ncomms15168 28447612 PMC5414183

[B47] Tokuyasu K. T. (1986). Application of cryoultramicrotomy to immunocytochemistry. *J. Microsc.* 143 139–149. 10.1111/j.1365-2818.1986.tb02772.x 3531524

[B48] White J. Johannes L. Mallard F. Girod A. Grill S. Reinsch S. (1999). Rab6 coordinates a novel Golgi to ER retrograde transport pathway in live cells. *J. Cell Biol.* 147 743–759. 10.1083/jcb.147.4.743 10562278 PMC2156170

[B49] Yu J. X. Guan Z. Nash H. A. (2006). The mushroom body defect gene product is an essential component of the meiosis II spindle apparatus in *Drosophila* oocytes. *Genetics* 173 243–253. 10.1534/genetics.105.051557 16510791 PMC1461445

[B50] Zerial M. McBride H. (2001). Rab proteins as membrane organizers. *Nat. Rev. Mol. Cell Biol.* 2 107–117. 10.1038/35052055 11252952

