# Supplementary figures and images for: GFP-Tagged Protein Detection by Electron Microscopy Using a GBP-APEX Tool in Drosophila
Source: Front Cell Dev Biol. 2021 Aug 12;9:719582. doi: 10.3389/fcell.2021.719582 (PMC8406855; doi:10.3389/fcell.2021.719582)

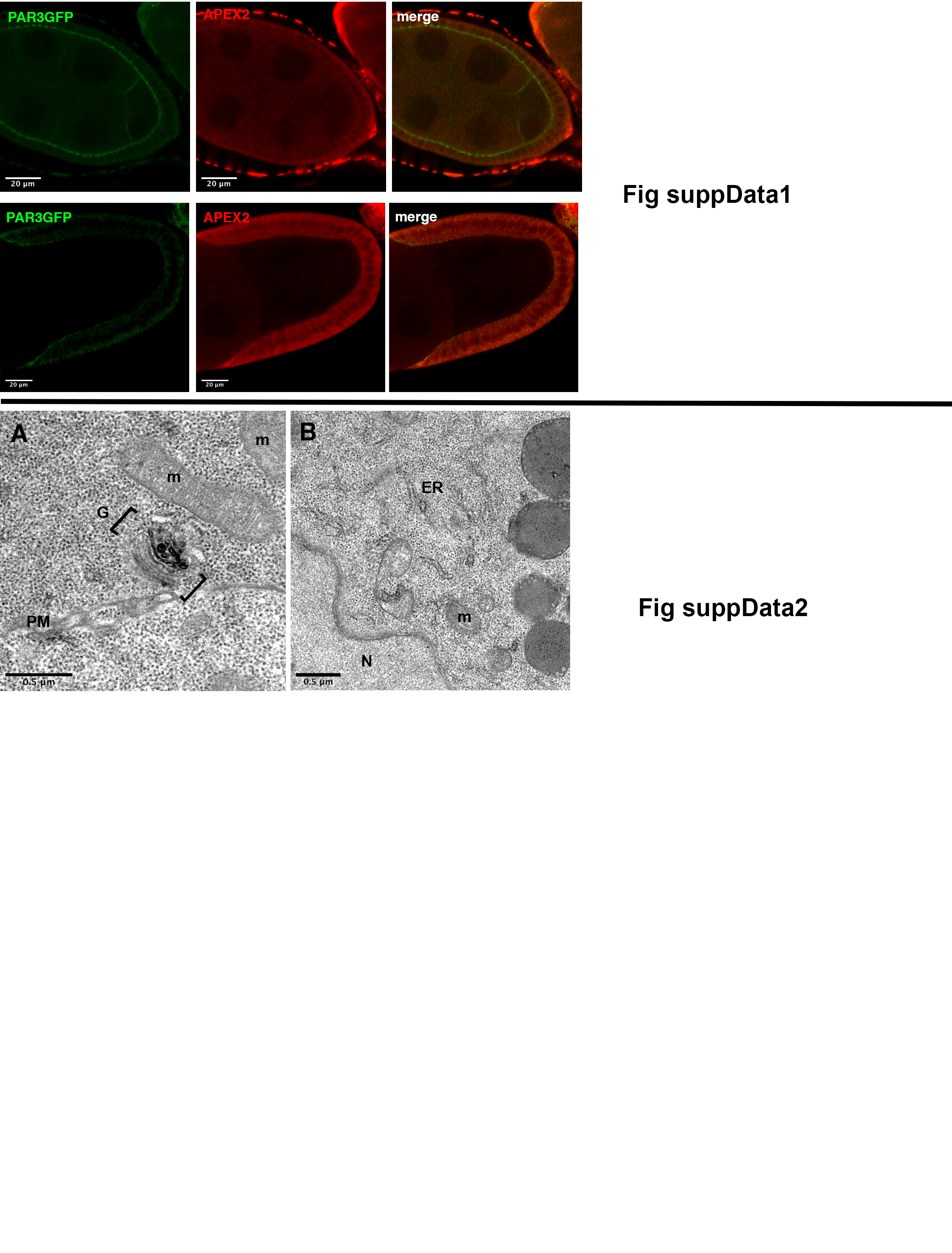

Supplement: Supplementary Figure 1 — Immunostaining anti-APEX2 on P(BazBAC.GFP); Tj-GAL4, UAS-APEX2-GBP ovarian follicles. Specific but diffuse staining is revealed in follicular cells. [file Image_1.TIF]
